# APACHE IV score in postoperative kidney transplantation

**DOI:** 10.5935/0103-507X.20180032

**Published:** 2018

**Authors:** Edison Moraes Rodrigues-Filho, Anderson Garcez

**Affiliations:** 1 Unidade de Terapia Intensiva de Transplantes, Hospital Dom Vicente Scherer, Irmandade da Santa Casa de Misericórdia de Porto Alegre - Porto Alegre (RS), Brasil.; 2 Rede Integrada de Pesquisa Institucional em Medicina Intensiva, Irmandade da Santa Casa de Misericórdia de Porto Alegre - Porto Alegre (RS), Brasil.; 3 Programa de Pós-Graduação em Saúde Coletiva, Universidade do Vale do Rio dos Sinos - São Leopoldo (RS), Brasil.

**Keywords:** Kidney transplantation, APACHE IV, Validation studies, Prognosis

## Abstract

**Objectives:**

To evaluate the calibration and discrimination of APACHE IV in the postoperative period after kidney transplantation.

**Methods:**

This clinical cohort study included 986 hospitalized adult patients in the immediate postoperative period after kidney transplantation, in a single center in southern Brazil.

**Results:**

Kidney transplant patients who died in hospital had significantly higher APACHE IV values and higher predicted mortality. The APACHE IV score showed adequate calibration (H-L 11.24 p = 0.188) and a good discrimination ROC curve of 0.738 (95%CI 0.643 - 0.833, p < 0.001), although SMR overestimated mortality (SMR = 0.73; 95%CI: 0.24 - 1.42, p = 0.664).

**Conclusions:**

The APACHE IV score showed adequate performance for predicting hospital outcomes in the postoperative period for kidney transplant recipients.

## INTRODUCTION

Transplantation is a widely used therapeutic modality for individuals with nephropathies in end-stage kidney disease.^(^^1^^)^ Although they frequently recover in an intensive care unit (ICU), this subgroup of patients has extremely low hospital mortality.^(^^2^^)^ The Acute Physiology and Chronic Health Evaluation (APACHE) is a frequently used score for the prediction of hospital death and for establishing benchmarking in ICU. APACHE is periodically updated from a predominantly North American database.^(^^3^^,^^4^^)^

Few studies have evaluated the performance of APACHE in the postoperative period after kidney transplant. Sawyer et al. evaluated the performance of APACHE II as a predictor of mortality in a cohort of 112 kidney transplant recipients, reporting mortality of 0%. In that study, APACHE II was not an adequate discriminator because it consistently overestimated mortality.^(^^2^^)^ Oliveira et al. in a retrospective cohort of 501 postoperative transplant recipients, of which 271 were kidney transplants, reported a mortality of less than 3%, confirming the low performance of APACHE II for this purpose.^(^^5^^)^ In a study using a recent APACHE score, APACHE IV, 224 kidney transplant recipients were included in the original validation cohort.^(^^6^^)^

Our study evaluated the APACHE IV as a predictor of mortality in the postoperative period after kidney transplant in southern Brazil. Our hypothesis was that APACHE IV could discriminate and calibrate adequately the prediction of hospital outcome in the postoperative period after renal transplants.

## METHODS

This was a clinical prospective and unicentric cohort study conducted in an 11-bed transplant ICU in southern Brazil (*Hospital Dom Vicente Scherer, Irmandade da Santa Casa de Misericórdia de Porto Alegre*). Patient data were entered on-site using a software program (Sistema Epimed Monitor, Epimed Solutions, Rio de Janeiro, Brazil).^(^^7^^)^ A single researcher collected the data for the purposes of the score. There were no missing data for the calculation of the score in any of the patients included in the study. There were no losses to follow-up. The APACHE IV score includes age, chronic health conditions and physiologic data, collected within the first 24 hours of ICU admission.^(^^6^^)^

All ICU patients in the immediate postoperative period receiving deceased donor organs and living donors ≥ 18 years were included from January 1, 2012 to December 31, 2016. Only the first admission to the ICU was considered for each patient. Conjugate kidney and pancreas transplants and kidney and liver were excluded.

Only the first ICU admission for each patient was used to predict hospital mortality within the same hospitalization. The APACHE IV score was calculated in the first 24 hours after ICU admission. The adjusted probability of hospital death, according to the diagnostic categories of APACHE IV, was also calculated.^(^^6^^)^

This study was approved by the Research Ethics Committee at *Irmandade Santa Casa de Misericórdia de Porto Alegre* (Plataforma Brasil CAAE number 19687113.8.2002.5335). The need for informed consent was waived since no intervention was required and no individual data were expected to be disclosed.

### Statistical analysis

The statistical analysis of the data was performed on the program Stata version 12.0 (StataCorp LP, College Station, Texas, USA). Descriptive statistics were used to describe the data, with calculation of mean, standard deviation, median and interquartile range, according to the distribution of variables. Student's *t*-test was used to evaluate the difference between means and the Mann-Whitney test to evaluate the distribution difference between medians, according to the normality of the distribution of the variables, as evaluated by the Kolmogorov-Smirnov test. To assess the discrimination and ability to classify survivors and non-survivors, discharge and death in the hospital were plotted on a receiver operating characteristic (ROC) curve and we calculated the respective area under receiver operator characteristic (AUROC) curve with its 95% confidence interval (95%CI) according to the APACHE IV score. The discrimination was considered to be excellent, very good, good, moderate and poor at AUROC values of 0.9 - 0.99, 0.8 - 0.89, 0.7 - 0.79, 0.6 - 0.69 and < 0.6, respectively. The quality of predictions was assessed by looking at the goodness-of-fit Hosmer-Lemeshow (H-L) test, evaluating the degree of calibration (degree of agreement between the predicted and observed death probability) across all the strata of probabilities of death. In this analysis, an H-L close to the degree of freedom, with equal to the number of categories minus 2 and a significance level greater than 5% (p > 0.05) indicated good calibration for the model. A calibration curve was constructed by plotting predicted mortality rates (x-axis) against observed mortality rates (y-axis), including grouped observations by deciles of predicted scale.

Standardized mortality ratios (SMR) with their respective 95%CI were calculated by dividing the observed mortality rate by the predicted mortality rate. An SMR equal to 1.0 indicated that the number of observed deaths equaled that of the expected number of deaths; an SMR >1.0 indicated occurrence of a greater number of deaths than expected.

## RESULTS

Of the total 986 patients, hospital mortality was 1.9%. During the study period, 1211 isolated adult kidney transplants were carried out. Therefore, data from 225 patients were not recorded because the postoperative period for these patients occurred in the recovery room (213) or another ICU in the hospital complex (12). The main reason for passing the postoperative period in the recovery room or in another ICU of the hospital complex was absence of bed availability in the ICU of the Dom Vicente Scherer Hospital.

There was a reduced rate of missing data. All clinical data were available. Regarding laboratory data, arterial blood gas analysis was not available for 272 (27.6%) patients and serum albumin was not available for 341 (34.6%) patients. Table 1 shows the characteristics of the study population.

**Table 1:** Characteristics of 986 patients who underwent kidney transplantation between 2012 and 2016

Characteristics	N (%)
Sex	
Male	600 (60.9)
Female	386 (39.1)
Donor	
Deceased	885 (89.76)
Living	101 (10.24)
Comorbidities	
Arterial hypertension	691 (70.08)
Diabetes	220 (22.31)
Previous myocardial infarction	94 (9.53)
Immunosuppression or steroid use	90 (9.13)
Previous stroke	48 (4.87)
Cardiac heart failure (according to NYHA)	23 (2.33)

NYHA - New York Heart Association.

There was only one case of readmission. Among the included patients, 43.6% required mechanical ventilation, 1.25% required vasopressors, and 61.7% required renal replacement therapy during ICU admission.

Table 2 shows the values of central tendency and dispersion for age, length of hospital stays and predicted mortality scores for hospital discharge and death outcomes obtained in this population of kidney transplant patients. Patients who died were older and had APACHE IV scores and predicted mortalities that were significantly higher.

**Table 2:** Central tendency and dispersion values for age, length of hospital stays, and mortality predicted for hospital discharge and death outcomes in kidney transplant patients

	Total sample	Hospital outcome		
		Discharge	Death	p value
Kidney transplant (N)	986	967	19	
Age (years)	48. ± 14.2	48.4 ± 14.2	58.0 ± 10.9	0.003
Hospital LOS (days)	23 (16 - 33)	23 (16 - 33)	24 (5 - 44)	0.835
ICU LOS (days)	2 (2 - 4)	2 (2 - 4)	3 (1 - 4)	0.683
APACHE IV (score)	55.0 ± 12.8	54.8 ± 12.6	66.7 ± 14.4	< 0.001
Predict mortality (%)	2.1 (1.4 - 3.2)	2.1 (1.4 - 3.2)	4.7 (2.7 - 5.6)	< 0.001

LOS - length of stay; ICU - intensive care unit. Results expressed in mean and standard deviation.

**Figure 1:**
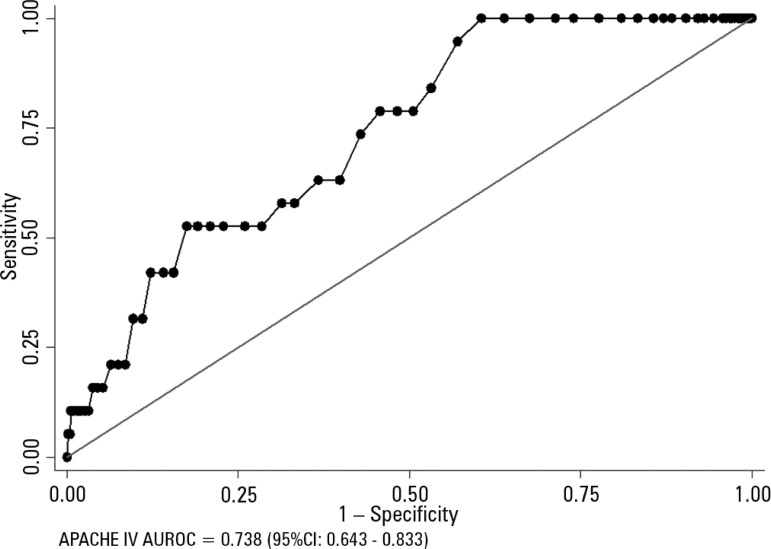
Analysis of the sensitivity and specificity for APACHE IV represented by the ROC curve (area under receiver operator curve - AUROC) in patients undergoing kidney transplantation, with death outcome in hospital. 95%CI - 95% confidence interval.

**Figure 2:**
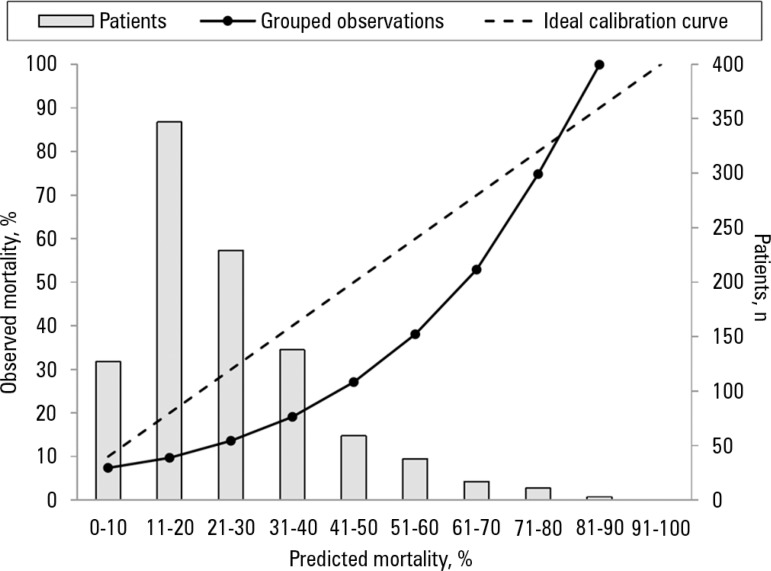
Calibration plot of predicted versus observed mortality in validation of APACHE IV score for hospital mortality in postoperative kidney transplant patients. Groups are deciles in predicted scale, and columns represent the number of patients in each stratum (10% of probability).

Figure 1 shows the sensitivity and specificity analysis for APACHE IV represented by the AUROC in patients undergoing kidney transplantation with hospital death outcome. Discrimination of the APACHE IV model showed good performance to predict in-hospital mortality after kidney transplantation, with an ROC curve of 0.738 (95%CI 0.643 - 0.833) p < 0.001. On calibration, the APACHE IV model performed adequately for in-hospital mortality (H-L 11.24 p = 0.188). The standardized mortality ratio overestimated the observed in-hospital mortality (SMR = 0.73; 95%CI 0.24 - 1.42, p = 0.664) (Figure 2).

## DISCUSSION

To the best of our knowledge, this was the first external validation of the APACHE IV score in postoperative kidney transplant patients. Kidney transplants have been showing a growing trend over the last 8 years in Brazil, especially secondary to the growth in the number of transplants with deceased donors.^(^^8^^)^ The Brazilian registry of transplants organized by the *Associação Brasileira de Transplante de Órgãos* (ABTO) counted 5,492 kidney transplants performed in Brazil in 2016.^(^^8^^)^ The existence of protocols for postoperative care and treatment of cardiovascular comorbidities in these patients has been a justification for the postoperative recovery in the intensive care unit. The variability in clinical care in intensive care, the high cost of care, the risk of death for critically ill patients and the possibility of comparing the performance of different units has led to the development and refinement of specific prognostic systems for the ICU.^(^^9^^)^

APACHE IV, the latest version of the APACHE system, has been little studied in our country. Nassar et al. compared the performance of APACHE IV, Mortality Probability Model (MPM) (0)-III and Simplified Acute Physiology Score (SAPS 3) scores in a population of 5,780 mixed critically ill patients.^(^^10^^)^ They showed that the three scores presented very good discrimination, but all models calibrated poorly and overestimated hospital mortality. Nassar et al published an external validation study, specifically in critically ill patients with acute coronary conditions.^(^^11^^)^

The kidney transplant patients who died in hospital were significantly older and had significantly higher APACHE IV scores. Predicted mortality was also significantly higher in those who died in the hospital. The APACHE IV score showed good discrimination and adequate calibration. In addition, the SMR overestimated the observed in-hospital mortality, although mortality in our population was higher than that described in the literature.^(^^2^^)^ Confirming our previous hypothesis, APACHE IV gave adequate performance for the prediction of hospital outcome in the postoperative period after kidney transplants.

Our discrimination and calibration results with APACHE IV diverged from those found with other scores, although the SMR overestimated the observed in-hospital mortality as did other scores. The APACHE II overestimated the mortality observed in the postoperative period after kidney transplant, but there was no detailed description of the discrimination and calibration procedures.^(^^2^^)^ APACHE II and SAPS III also overestimated the postoperative mortality of renal transplant patients with moderate discrimination and inadequate calibration.^(^^5^^)^

Our findings were relatively unexpected. General prognostic models usually do not perform well in specific subgroups of patients because they may be under-represented in the developed cohort. For some specific diagnoses, a specific prognostic model may be an attractive alternative.^(^^11^^)^ The use of scores that integrate donor and recipient information is a trend in the evaluation of solid organ transplants.^(^^12^^)^

In our study, despite good discrimination and adequate calibration, SMR overestimated mortality by APACHE IV. The SMR, due to its simplicity, has been used as a benchmark both to compare the performance of the same unit over time and to compare the performance of different units.^(^^13^^)^ Together with the standardized resource utilization ratio, the SMR makes up the efficiency matrix that allows the comparison of different units, classifying them in various performance quadrants.^(^^14^^)^ However, the SMR should be interpreted with caution, especially in units with diagnoses as specific as ours, a unit specialized in the critical care of organ and tissue transplants. Thus, the overestimation or underestimation detected in the SMR reflects the fact that this measure is global and does not separately consider the mortality in the various severity strata. In fact, in our study, the overestimation of mortality by the SMR assessment appeared to occur in the less risky deciles, especially in the 1^st^, 2^nd^ and 4^th^ decile of severity, in which no deaths were observed among our transplanted patients. This overestimation in the strata of lower mortality was probably due to the score of several variables that reflected the high rate of primary graft dysfunction among donor recipients dying in our country.^(^^15^^)^ These variables included serum creatinine, BUN (blood urea nitrogen), bicarbonate, potassium and sodium, in addition to 24-hour diuresis. In 2016, for example, with only deceased donors in the sample, the need for hemodialysis in the first 24 hours after transplantation reached 73.9%.

The strongest aspect of our study was the large sample analyzed. Another important aspect was the possibility of prediction of outcome with a score that can be incorporated into the routine of the ICU without the needs of donor and transoperative data. However, our study had several weaknesses: it was a single-center study that used an administrative database. The amount of missing data was low, but the absence of arterial blood gas analysis may have underestimated the predicted mortality, since patients in the postoperative period after kidney transplantation have metabolic acidosis consequent to inadequate preoperative dialysis. These missing data could have reduced the SMR and influenced the calibration, but in an inverse sense to that previously reported for SAPS 3.^(^^16^^)^ These weaknesses limit the external validity of our findings. Another weakness of our study was the evaluation of a population with low hospital mortality, possibly limiting the findings obtained with the APACHE IV score. In this group of patients, other outcomes, such as delayed graft function defined by the need for dialysis in the first week post-transplantation, may be more useful clinically.

## CONCLUSIONS

With the available data, it was possible to consider the use of APACHE IV for the prediction of hospital death in the postoperative period after kidney transplantation. However, to establish benchmarking, APACHE IV may be limited in our setting, because of overestimation of mortality among patients at lower risk.
